# Association of triglyceride-glucose index and delirium in patients with sepsis: a retrospective study

**DOI:** 10.1186/s12944-024-02213-x

**Published:** 2024-07-25

**Authors:** Yipeng Fang, Aizhen Dou, Yuehao Shen, Tianyu Li, Haiying Liu, Yan Cui, Keliang Xie

**Affiliations:** 1 Department of Critical Care Medicine, Tianjin Institute of Anesthesiology, Tianjin Medical University General Hospital, 154th Anshan Road, Tianjin, 300052 China; 2 Department of Anesthesiology, Tianjin Institute of Anesthesiology, Tianjin Medical University General Hospital, Tianjin, 300052 China; 3 Department of Pathogen Biology, School of Basic Medical Sciences, Tianjin Medical University, No. 22, Qixiangtai Road, Heping District, Tianjin, 300070 China

**Keywords:** Sepsis, Delirium, Triglyceride-glucose index, TyG index, Insulin resistance

## Abstract

**Objective:**

It is well known that glucose and lipid metabolism disorders and insulin resistance are common in sepsis, which affect the occurrence and prognosis of multiple organ dysfunction in septic patients. Previous study reported the predictive value of triglyceride-glucose index (TyG), a clinical indicator for insulin resistance, in postoperative delirium patients. However, it remains unclear whether the TyG index is a novel predictive biomarker for sepsis-associated delirium. The aim of this study is to explore the relationship between TyG index and the risk of delirium in patients with sepsis.

**Methods:**

Adult septic patients were identified from the MIMIC-IV database and divided into four groups based on the mean value of TyG. The primary outcome was the incidence of delirium. The association between TyG and the risk of developing delirium was evaluated by restricted cubic spline (RCS), multivariate logistic regression and subgroup analysis. Propensity Score Matching (PSM) method was used to balance the baseline data.

**Results:**

A total of 3,331 septic patients were included in the analysis, and further divided into four groups: Q1 (TyG ≤ 8.67), Q2 (8.67 < TyG ≤ 9.08), Q3 (9.08 < TyG ≤ 9.61), and Q4 (TyG > 9.61). The RCS curves demonstrated a non-linear positive relationship between TyG index and the risk of developing delirium, and an optimal cut-of value 9.09 was recommended. After balancing the baseline information by PSM, patients in the TyG > 9.09 group had a significant higher incidence of delirium compared with those in the TyG ≤ 9.09 group. In logistic regression analysis, TyG > 9.09 was significantly associated with lower risk of developing delirium in both original cohort (OR 1.54–1.78, all *P* < 0.001) and the PSM cohort (OR 1.41–1.48, all *P* < 0.001). No association was found between the TyG index and mortality (all *P* > 0.05). In subgroup analysis, our findings were consistent (all OR > 1 in all subgroups).

**Conclusion:**

Our study demonstrated an independent association between TyG index and increased risk of delirium in septic patients, indicating that TyG index can serve as a biomarker for delirium in sepsis.

**Supplementary Information:**

The online version contains supplementary material available at 10.1186/s12944-024-02213-x.

## Background

In the current consensus definition (Sepsis 3.0), sepsis is defined as the host dysregulated response to infection, leading to life-threatening multiple organ dysfunction [1]. It is recognized as a global health challenge, bringing a significant disease burden to many countries [2]. A previous study showed that the incidence rate of sepsis was 437/100,000 per year, the in-hospital mortality rate was 17%, and the mortality rate of severe sepsis was 26% [3]. Sepsis remains the prime mortality reason in intensive care units (ICUs) all over the world [4]. And the high mortality rate was mainly caused by the adverse prognosis of multiple organ dysfunction induced by sepsis. Therefore, early identification and prevention of sepsis and correlative organ dysfunction can significantly improve the prognosis for patients, and reduce mortality and disability.

Delirium is an acute brain dysfunction characterized by drastic changes in cognitive ability, especially in attention, consciousness and impaired arousal, which can be triggered by various underlying factors, including acute illness, drug-taking or withdrawal reaction, trauma, and surgery [5]. Sepsis is one of the most important and strongest risk factors in the ICU departments [6–8]. The neurological symptoms that occur in patients with sepsis are called sepsis associated encephalopathy (SAE), and delirium is the main early symptom [9]. Previous studies have shown that delirium can have a maximum impact on up to 70% of septic patients [10]. SAE not only increases the mortality of patients with sepsis, but also leads to the long-term cognitive impairment and psychological disorders [11]. It has been reported that up to 51% of septic patients do not return to their full-time employment 1 year after their occurrence of sepsis [12], the risk of suicide is significantly increased within the first two years after recovery from sepsis [13]. It can be explained by the hippocampal and frontal lobe damage associated with SAE [14, 15]. Therefore, prospective biomarkers are needed in clinical practice to predict the risk of delirium in septic patients and to intervene early.

Insulin resistance (IR) is a metabolic disease characterized by impaired response of target tissues to insulin, leading to abnormal glucose and lipid metabolism [16–18]. When sepsis occurs, a stress response occurs with the activation of the sympathetic nervous system and increased adrenal cortex hormones, causing disorder in glucose metabolism, even IR in severe patients [19–21]. In addition, the widespread release of pro-inflammatory factors and imbalanced oxidative stress may be another major reason for inducing IR for sepsis [22]. Acute blood glucose fluctuations have been reported to increase the risk of mortality in patients with sepsis [23]. At present, the gold standard for evaluating IR is high insulin glucose clamp (HEC), which is suitable for various types of people, but disadvantaged from complex operating techniques, high cost, and invasiveness [24, 25]. More widely used is the Homeostasis Model Assessment-Insulin Resistance (HOMA-IR) index, but it is also restricted by individuals receiving insulin therapy or those with non-functionality β individual cells [26]. There have been studies proposing the triglyceride-glucose (TyG) index as a simple alternative biomarker for IR, which has been proven to predict the prognosis of cardiovascular diseases, including coronary heart disease, heart failure, acute myocardial infarction, stroke, and hypertension [27–29].

A study suggested that the TyG index had certain value in early detection of IR in septic patients and predicting the increased risk of all-cause in-hospital mortality [22]. The specific pathophysiology of delirium is still unclear, but metabolic disorders are thought to be an important factor. Shinohara M et al. reported that the bidirectional association between diabetes and cognitive dysfunction could be mediated by IR and the imbalance of blood glucose metabolism [30]. In addition, Miao Sun et al. pointed out that TyG index is a new biomarker to predict postoperative delirium (POD) in elderly patients with type 2 diabetes [31]. These have proved a close association between the occurrence of delirium and IR. However, it is still indistinct whether the TyG index can predict the occurrence of delirium in patients with sepsis, which has been reported to impact on up to 70% of patients with sepsis [10].

The purpose of this study is to explore the association between the TyG index and the risk of developing delirium, which may reveal a new predictive biomarker for early intervention of delirium in septic patients. These issues will affect the development of more effective prevention and treatment strategies for sepsis-associated delirium in clinical practice.

## Materials and methods

### Data sources and study design

This was a single-center retrospective clinical study, with data derived from the Medical Information Mart for Intensive Care (MIMIC-IV version 2.2) database [32]. MIMIC-IV version 2.2 is the latest version of the MIMIC database, constructed by the Massachusetts Institute of Technology (MIT). The data comprised over 70,000 records admitted to the ICU departments at the Beth Israel Deaconess Medical Center (BIDMC) between 2008 and 2019. The author, FYP, obtained database access permission (No. 43,025,968) through the National Institutes of Health (NIH) web-based course training and examination. The MIMIC-IV database has received ethical approval from the Institutional Review Boards of the MIT and BIDMC. Informed consent can be waived for anonymized personal information. The present study did not require any additional data, therefore no further ethical approval or informed consent is required.

### Inclusion and exclusion criteria

Inclusion criteria were as follows: adult patients (age ≥ 18 years) with a definite diagnosis of sepsis during hospitalization according to the Sepsis 3.0 definition [1], and documented serum levels of triglyceride and glucose from their admission until their diagnosis of delirium. Patients who met the following criteria were excluded from the final analysis: non-first ICU admission records for patients with repeated hospitalizations; the presence of dementia, which may affect the assessment of delirium; presence of delirium before the onset of sepsis; ICU stay of less than 24 h.

### Exposure and outcome

The primary exposure for the present study was the TyG index, which is a composite index composed of triglyceride and glucose, calculated using the following formula: In [serum triglyceride(mg/dl) × serum blood glucose(mg/dl)/2] [33]. We retained the triglyceride and glucose results for all patients from their admission until their diagnosis of delirium. We further calculated the TyG index using the maximum, minimum, initial, and mean values of both triglyceride and glucose.

The primary outcome of our study was the novel occurrence of delirium after the diagnosis of sepsis. Delirium assessment mainly followed the widely used clinical delirium assessment tool, the Confusion Assessment Method for the ICU (CAM-ICU) [11]. It has been reported that CAM-ICU has a pooled sensitivity of 81% (95% CI: 57–93%) and a pooled specificity of 98% (95% CI: 86–100%) for delirium screening, making it suitable for accurate diagnosis of delirium [11]. Secondary outcome measures included 28-day mortality, 90-day mortality, hospital mortality, ICU mortality, total length of hospital stay (Hospital-LOS), and length of ICU stay (ICU-LOS).

### Data extraction

The author (FYP) was responsible for data extraction using PostgreSQL and PgAdmin4 software. The baseline information included in this study comprised demographic characteristics, comorbidities, laboratory parameters, special interventions received, and disease severity scores. The demographic characteristics included age, sex, race and body weight on the admission. The comorbidities, including cerebral infarction, cerebral hemorrhage, coronary heart disease, heart failure, atrial fibrillation, hypertension, diabetes mellitus, anemia, chronic pulmonary disease, liver disease, chronic kidney disease, malignant cancer, were documented. The initial results of laboratory parameters during their ICU stay, such as white blood cell, hemoglobin, platelet, sodium, potassium and creatinine, were collected. The records of receiving special interventions, including mechanical ventilation, vasoactive drug and midazolam, were extracted. The maximum of SOFA score was used to present the severity of the disease. For special interventions, and SOFA score, only data before the development of delirium was included in the final analysis.

### Data cleaning

When triglycerides or blood glucose data were lacking to calculate the TyG value, patients were excluded from the final cohort. For continuous variables with a normal distribution, data exceeding 3-fold standard deviation (SD) from the mean value were considered as outliers. For continuous variables with non-normal distributions, data exceeding 3-fold of the interquartile range (IQR) above the third quartile (Q3) or below the first quartile (Q1) were considered as outliers. All outliers were treated as missing values. Since the missing proportion of continuous variables in all baseline data was less than 5%, we used the mean or median to impute missing data according to their distribution characteristics.

### Statistical analysis

During baseline analysis, patients were divided into two groups according to whether developing delirium during their ICU stay. Continuous variables with a normal distribution were presented as mean ± SD, and analyzed using Student’s t-test. Continuous variables with a non-normal distribution were represented by the median and IQR, and further analyzed using the Mann-Whitney U test. For the analysis of continuous variables among multiple groups, one-way ANOVA and the Kruskal-Walli’s test was used. Categorical variables were expressed as counts and percentages and analyzed using the Chi-square test, and the *P*-values was further adjusted by the Bonferroni method in multiple group comparisons.

All patients were further divided into four categories according to their IQR for TyG mean values, including the Q1(TyG ≤ 8.67), Q2(8.67 < TyG ≤ 9.08), Q3(9.08 < TyG ≤ 9.61) and Q4(TyG > 9.61) categories, to further explore the relationship between different TyG categories and outcome indicators. Additionally, we utilized restricted cubic spline (RCS) regression to visually present the potential nonlinear relationship between TyG and the risk of delirium. We set 4 knots, with their positions set to the TyG values corresponding to quantiles 0.05, 0.35, 0.65 and 0.95, which were 8.07, 8.84, 9.35 and 10.36 respectively.

To eliminate the impact of confounding factors on the outcomes, we performed Propensity Score Matching (PSM) for sensitivity analysis to adjust for the confounders between the groups [34]. We calculated the propensity scores using a multivariable logistic regression model. The included variables were demographic characteristics (age, sex, race, body weight), comorbidities (cerebral infarction, cerebral hemorrhage, coronary heart disease, heart failure, atrial fibrillation, hypertension, diabetes mellitus, anemia, chronic pulmonary disease, liver disease, chronic kidney disease, malignant cancer), laboratory parameters (white blood cell, hemoglobin, platelet, sodium, potassium and creatinine), special interventions received (mechanical ventilation, vasoactive drug and midazolam) and disease severity scores (SOFA and SAPSII). Based on a TyG index cut-off of 9.09, we divided the patients into two groups (TyG ≤ 9.09 & TyG > 9.09), and further performed 1:1 matching using nearest neighbor matching with a caliper width of 0.02 without replacement. The final PSM matched cohort comprised 1,119 patients with delirium and 1,119 patients without delirium.

In addition, the logistic regression analysis models were used to further determine the predictive value of the TyG ratio for the risk of developing delirium. To reduce the effect of potential confounders on the outcome, we sequentially included different variables in the logistic regression models and constructed different adjusted models. Adjusted model 1 included patient demographics and comorbidities. Adjusted model 2 included laboratory results in the analysis. Adjusted model 3 additionally considered the effect of factors such as mechanical ventilation, vasopressor use, midazolam administration, and severity of illness scores on outcomes. Multicollinearity between variables was assessed by variance inflation factor (VIF), with confounders with VIF > 10 converted to binary categorical variables based on median or mean to address multicollinearity issues (see Tables S1-S2). These adjusted models were also used in the RCS analysis.

Based on the logistic regression analysis and the constructed adjusted model 3, subgroup analysis was further performed to verify the robustness of our findings and to explore the potential interactive factors. For subgroup analysis, patients were divided into two opposing subgroups based on age, sex, race, body weight, severity of illness score, intracerebral hemorrhage, intracerebral ischemia, diabetes, usage of mechanical ventilation and usage of vasopressors. For continuous variables, age and SOFA score, grouping was performed according to mean and median.

Statistical analysis was performed using Stata software (version 15.0 SE) and R language (version 4.3.2), with a bilateral *P*-value ≤ 0.05 as the basis for statistically significant differences.

## Results

### Baseline information and clinical endpoints

**Fig. 1:**
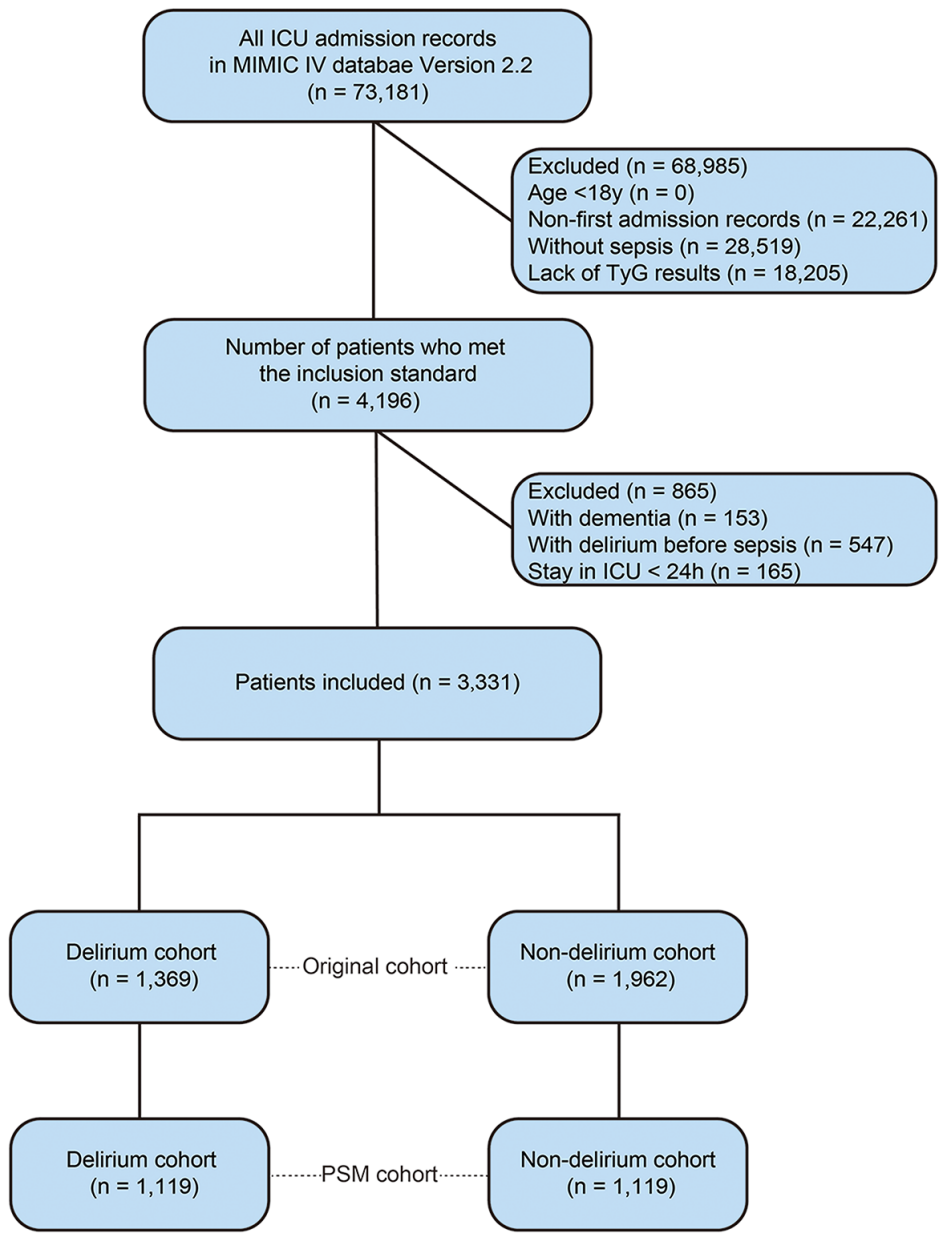
The flow chart of present study

Figure 1 depicts the flowchart of the cohort selection. A total of 3,331 patients were included in the final analysis, including 1,369 delirium patients and 1,962 non-delirium patients. Table 1 shows the comparative baseline information between the delirium and non-delirium groups. We found that patients in the delirium group were younger, less likely to be Caucasian/white, and heavier (all *P* < 0.01). The delirium group had a higher proportion of patients with comorbidities including cerebral infarction, anemia and liver disease, but less coronary heart disease (all *P* < 0.001). Patients with delirium were more likely to have received mechanical ventilation and vasoactive drugs, but less likely to have received midazolam before developing delirium (all *P* < 0.001). Severity of illness scores, such as SOFA and SAPSII, were higher in the delirium group (all *P* < 0.001). When comparing the exposure indicator, we found that the initial (9.04 ± 0.75 vs. 9.24 ± 0.84, *P* < 0.001), maximum (9.53 ± 0.76 vs. 9.65 ± 0.84, *P* < 0.001), minimum (8.49 ± 0.69 vs. 8.80 ± 0.75, *P* < 0.001) and mean (9.05 ± 0.66 vs. 9.27 ± 0.73, *P* < 0.001) values of the TyG ratio were significantly higher in delirium patients than in non-delirium patients. The delirium group had higher mortality rates and longer LOS than the non-delirium group (all *P* < 0.01).

**Table 1:** Comparison of the baseline information and clinical endpoints in septic patients with and without delirium

Variable	Overall	Non-delirium	Delirium	*P*-value
Number	3,331	1,962	1,369	
Age (years)	62.78 ± 16.33	63.66 ± 16.28	61.51 ± 16.33	< 0.001
Male (%)	2,043(61.33)	1,195(60.91)	848(61.94)	0.546
Ethnicity, white (%)	2,045(61.39)	1,304(66.46)	741(54.13)	< 0.001
Weight (kg)	86.10 ± 26.13	85.01 ± 25.65	87.66 ± 26.75	0.004
Comorbidity				
Cerebral infarction (%)	560(16.81)	289(14.73)	271(19.80)	< 0.001
Cerebral hemorrhage (%)	212(6.36)	121(6.17)	91(6.65)	0.577
Coronary heart disease (%)	884(26.54)	606(30.89)	278(20.31)	< 0.001
Heart failure (%)	908(27.26)	543(27.68)	365(26.66)	0.518
Hypertension (%)	1,377(41.34)	823(41.95)	554(40.47)	0.394
Diabetes mellitus (%)	1,009(30.29)	597(30.43)	412(30.09)	0.837
Atrial fibrillation (%)	977(29.93)	586(29.87)	411(30.02)	0.924
Anemia (%)	1,648(49.47)	871(44.39)	777(56.76)	< 0.001
Chronic pulmonary disease (%)	845(25.37)	486(24.62)	362(26.44)	0.234
Chronic kidney disease (%)	644(19.33)	384(19.57)	260(18.99)	0.677
Liver disease (%)	740(22.22)	388(19.78)	352(25.71)	< 0.001
Malignant cancer (%)	489(14.68)	304(15.49)	185(13.51	0.112
Laboratory parameter				
White blood cell (k/uL)	12.1(8.4,16.7)	11.9(8.3,16.4)	12.5(8.7,17.2)	0.030
Hemoglobin (g/dL)	10.77 ± 2.39	10.77 ± 2.28	10.75 ± 2.54	0.766
Platelets (k/uL)	188(128,253)	191(133,258)	183(121,249)	0.002
Sodium (mmol/L)	138.28 ± 5.50	138.24 ± 5.26	138.33 ± 5.82	0.641
Potassium (mmol/L)	4.23 ± 0.76	4.18 ± 0.72	4.30 ± 0.82	< 0.001
Creatinine (mg/dl)	1.5(1.0,2.9)	1.4(0.9,2.4)	1.7(1.0,3.5)	< 0.001
Intervention				
Mechanical ventilation (%)	2,331(69.98)	1,224(62.39)	1,107(80.86)	< 0.001
Vasoactive drug (%)	1,447(43.44)	736(37.51)	711(51.94)	< 0.001
Midazolam exposure (%)	1,252(37.59)	812(41.39)	440(32.14)	< 0.001
Disease severity score				
SOFA score	8(5,11)	7(4,10)	10(7,13)	< 0.001
SAPSII score	40(31,51)	38(30,48)	43(34,54)	< 0.001
TyG index				
Initial value	9.12 ± 0.80	9.04 ± 0.75	9.24 ± 0.84	< 0.001
Maximum value	9.58 ± 0.80	9.53 ± 0.76	9.65 ± 0.84	< 0.001
Minimum value	8.62 ± 0.73	8.49 ± 0.69	8.80 ± 0.75	< 0.001
Mean value	9.13 ± 0.70	9.05 ± 0.66	9.27 ± 0.73	< 0.001
Outcomes				
Hospital mortality (%)	711(21.34)	376(19.16)	335(24.47)	< 0.001
ICU mortality (%)	545(16.36)	282(14.37)	263(19.21)	< 0.001
28-day mortality (%)	736(22.10)	401(20.44)	335(24.47)	0.006
90-day mortality (%)	967(29.03)	535(27.27)	432(31.56)	0.007
Hospital LOS (days)	15.7(9.0,25.7)	13.2(7.9,22.7)	19.1(11.7,28.7)	< 0.001
ICU LOS (days)	6.2(3.0,12.4)	4.2(2.3,9.1)	9.2(5.4,15.6)	< 0.001

Tip Continuous variables are displayed as mean (standard deviation) or median (first quartile–third quartile); categorical variables are displayed as count (percentage); ICU, intensive care unit; LOS, length of stay; SAPS, Simplified Acute Physiology Score, SOFA, Sequential Organ Failure Assessment; TyG, triglyceride-glucose index

### Clinical outcomes in different TyG categories

After grouping according to mean TyG values, significant differences in the incidence of delirium were observed between the different categories (*P* < 0.001, Table 2). The incidence of delirium was similar between the Q1 and Q2 groups, but the incidence of delirium increased significantly with further increases in TyG. The incidence of delirium in the Q4 category was as high as 54.58%. There were no significant differences in mortality rates between the different categories (all *P* > 0.05). With increasing TyG levels, both hospital-LOS and ICU-LOS gradually increased (all *P* < 0.001). The shortest durations were observed in Q1, with hospital-LOS of 13.6 days (8.0, 23.2) and ICU-LOS of 4.6 days (2.5, 9.2). The longest durations were observed in Q4, with hospital-LOS of 17.5 days (10.5, 27.7) and ICU-LOS of 9.1 days (4.5, 15.7).

**Table 2:** Clinical outcome for patients in different TyG categories

	Overall	Q1(TyG ≤ 8.67)	Q2(8.67 < TyG ≤ 9.08)	Q3(9.08 < TyG ≤ 9.61)	Q4(TyG > 9.61)	*P*- value
Number	3,331	825	838	838	830	
Delirium (%)	1369(41.10)	281(34.06)	285(34.01)	350(41.77)	453(54.58)	< 0.001
28-day mortality (%)	736(22.10)	185(22.42)	176(21.00)	174(20.6)	201(24.22)	0.300
90-day mortality (%)	967(29.03)	249(30.18)	238(28.40)	217(25.89)	263(31.69)	0.057
hospital mortality (%)	711(21.34)	167(20.24)	167(19.93)	172(20.53)	205(24.70)	0.058
ICU mortality (%)	545(16.36)	119(14.42)	129(15.39)	136(16.23)	161(19.40)	0.038
Hospital LOS (days)	15.7(9.0,25.7)	13.6(8.0,23.2)	15.1(9.0,23.7)	16.6(9.0,27.1)	17.5(10.5,27.7)	< 0.001
ICU LOS (days)	6.2(3.0,12.4)	4.6(2.5,9.2)	5.7(2.8,10.9)	6.2(2.9,12.6)	9.1(4.5,15.7)	< 0.001

Tip Continuous variables are displayed as mean (standard deviation) or median (first quartile–third quartile); categorical variables are displayed as count (percentage); ICU, intensive care unit; LOS, length of stay; TyG, triglyceride-glucose ratio

### Non-linear relationship between TyG and the risk of delirium

**Fig. 2:**
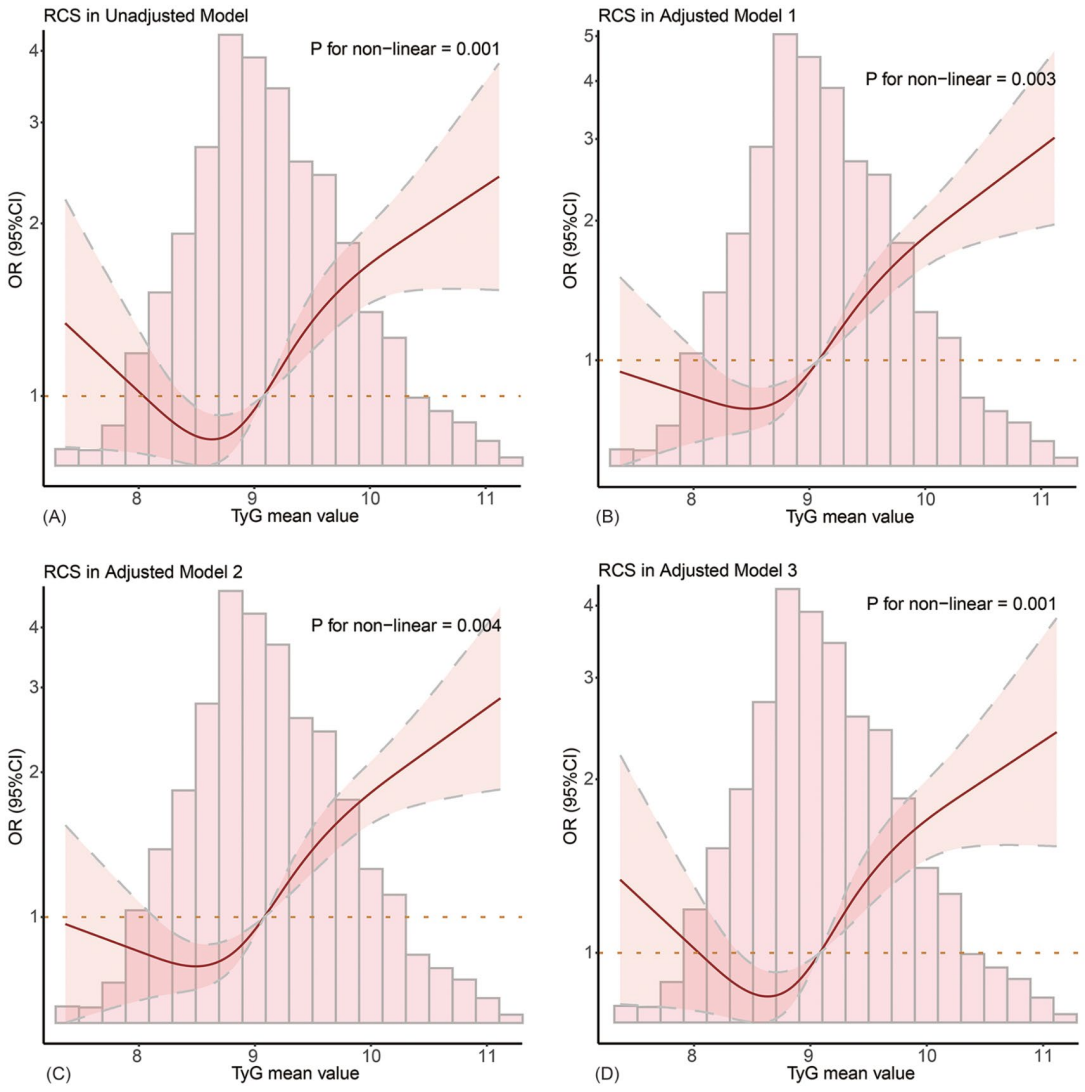
Non-linear relationship between TyG index and the risk of developing delirium. (**A**) presents the result obtained from the unadjusted model, while (**B**-**D**) shown the findings in the adjusted models. The red solid line represents odd ratio (OR), the gray dashed lines represent the upper and lower 95% confidence interval (95%CI), the red area represents the area of CI, and the horizontal dashed line represents the invalid line (OR = 1). The bar chart represents the proportion of patient distribution

Figure 2 shows the relationship between mean TyG values and the risk of developing delirium using RCS curves. The curve shows a J-shaped non-linear relationship between the two (*P* for non-linear < 0.001). The effect of low TyG values on delirium is not significant, but as TyG values increase, the risk of developing delirium increase significantly, with an optimal cut-off value of 9.09 (Fig. 2A), suggesting that TyG values above 9.09 was significantly associated with the increased risk of the development of delirium in septic patients. This J-shaped non-linear relationship persisted across different adjusted models (Fig. 2B and D).

### Propensity score matching

In the PSM-matched cohort, all baseline characteristics were balanced (all *P* > 0.05), except for creatinine levels (see Table 3). The creatinine level was significantly higher in the TyG > 9.09 group than in the TyG ≤ 9.09 group (1.5 [1.0, 2.9] vs. 1.4 [0.9, 2.7], *P* = 0.020). The incidence of delirium was higher in the TyG > 9.09 group compared to the TyG ≤ 9.09 group (44.86% vs. 36.64%, *P* < 0.001). There was no difference in mortality between the two groups (all *P* > 0.05). Patients in the TyG > 9.09 group had a longer Hospital- LOS (16.8 [9.7, 27.7] vs. 15.1 [8.8, 25.0], *P* = 0.002) and a longer ICU-LOS (7.1 [3.3, 13.3] vs. 6.0 [3.0, 11.6], *P* = 0.003). The RCS curve reveals that the J-shaped association between the TyG value and the risk of developing delirium in septic patients was still present in the PSM cohort (shown in Figure S1).

**Table 3:** Baseline and clinical endpoints in PSM cohorts

Variable	Overall	TyG ≤ 9.09	TyG > 9.09	*P*-value
Number	2,238	1,119	1,119	
Age (years)	61.04 ± 15.91	61.86 ± 16.30	62.21 ± 15.51	0.609
Male (%)	1,372(61.30)	705(63.00)	667(59.61)	0.099
Ethnicity, white (%)	1,373(61.35)	678(60.59)	695(62.11)	0.461
Weight (kg)	85.26 ± 24.32	85.18 ± 26.31	85.34 ± 22.16	0.879
Comorbidity				
Cerebral infarction (%)	358(16.00)	182(16.26)	176(15.73)	0.729
Cerebral hemorrhage (%)	128(5.72)	64(5.72)	64(5.42)	> 0.999
Coronary heart disease (%)	572(25.56)	289(25.83)	283(25.29)	0.771
Heart failure (%)	585(26.14)	290(25.92)	295(26.36)	0.810
Hypertension (%)	928(41.47)	464(41.47)	464(41.47)	> 0.999
Diabetes mellitus (%)	642(28.69)	334(29.85)	308(27.52)	0.224
Atrial fibrillation (%)	635(28.37)	334(29.85)	301(26.90)	0.122
Anemia (%)	1,106(49.42)	548(48.97)	558(49.87)	0.672
Chronic pulmonary disease (%)	545(24.35)	269(24.04)	276(24.66)	0.730
Chronic kidney disease (%)	401(17.92)	202(18.05)	199(17.78)	0.869
Liver disease (%)	507(22.35)	253(22.31)	254(22.40)	0.960
Malignant cancer (%)	345(15.42)	173(15.46)	172(15.37)	0.953
Laboratory parameter				
White blood cell (k/uL)	11.9(8.3,16.6)	11.9(8.3,16.4)	11.9(8.3,16.6)	0.963
Hemoglobin (g/dL)	10.78 ± 2.39	10.81 ± 2.40	10.76 ± 2.39	0.611
Platelets (k/uL)	185(122,251)	184(123,248)	185(121,254)	0.831
Sodium (mmol/L)	138.28 ± 5.49	138.23 ± 5.63	138.33 ± 5.34	0.653
Potassium (mmol/L)	4.21 ± 0.74	4.21 ± 0.76	4.21 ± 0.73	0.803
Creatinine (mg/dl)	1.5(1.0,2.7)	1.4(0.9,2.7)	1.5(1.0,2.9)	0.020
Intervention				
Mechanical ventilation (%)	1,606(71.76)	806(72.03)	800(71.49)	0.778
Vasoactive drug (%)	1,000(44.68)	507(45.31)	493(44.06)	0.552
Midazolam exposure (%)	849(37.94)	428(38.25)	421(37.62)	0.760
Disease severity score				
SOFA score	8(5,11)	8(5,12)	8(5,11)	0.989
SAPSII score	40(31,50)	40(31,51)	40(31,50)	0.393
Outcomes				
Delirium (%)	912(40.75)	410(36.64)	502(44.86)	< 0.001
Hospital mortality (%)	492(21.98)	254(22.70)	238(21.27)	0.414
ICU mortality (%)	373(16.67)	195(17.43)	178(15.91)	0.335
28-day mortality (%)	493(22.03)	255(22.79)	238(21.37)	0.386
90-day mortality (%)	646(28.87)	338(30.21)	308(27.52)	0.162
Hospital LOS (days)	15.8(9.1,26.7)	15.1(8.8,25.0)	16.8(9.7,27.7)	0.002
ICU LOS (days)	6.4(3.1,12.7)	6.0(3.0,11.6)	7.1(3.3,13.3)	0.003

Tip Continuous variables are displayed as mean (standard deviation) or median (first quartile–third quartile); categorical variables are displayed as count (percentage); ICU, intensive care unit; LOS, length of stay; SAPS, Simplified Acute Physiology Score, SOFA, Sequential Organ Failure Assessment; TyG, triglyceride-glucose index. A multivariate logistic regression model was used to estimate the patient’s propensity scores. A 1:1 nearest neighbor match without replacement was applied with a caliper width of 0.02

### Logistic regression analysis

The results of the logistic regression analysis are shown in Table 4. It was found that TyG value > 9.09 was significantly associated with the development of delirium, with an OR (95% CI) of 1.78(1.55–2.04, *P* < 0.001). After adjusting for potential confounding factors, the significant association between increased TyG value (> 9.09) and the risk of developing delirium still existed (OR 1.54–1.73 in different models, all *P* < 0.001). After balancing the baseline by PSM, TyG value > 9.09 was still significantly associated with the increased risk of developing delirium, which could increase the risk by 41–48% in different models (OR 1.41–1.48, all *P* < 0.001).

**Table 4:** Logistic regression analysis detecting the value of TyG > 9.09 on developing delirium in septic patients

	Original cohort		PSM cohort	
	OR (95% CI)	*P*-value	OR (95% CI)	*P*-value
Unadjusted Model	1.78(1.55–2.04)	< 0.001	1.41(1.19–1.67)	< 0.001
Adjusted Model 1	1.73(1.49–2.02)	< 0.001	1.43 (1.20–1.70)	< 0.001
Adjusted Model 2	1.70(1.46–1.98)	< 0.001	1.42 (1.20–1.70)	< 0.001
Adjusted Model 3	1.54(1.31–1.82)	< 0.001	1.48 (1.23–1.78)	< 0.001

Model 1 = adjusting sex, age, ethnicity, weight and comorbidities (cerebral infarction, cerebral hemorrhage, sepsis, hypertension, coronary heart disease, heart failure, diabetes, chronic kidney disease, chronic pulmonary disease, liver disease, anemia, malignant cancer)

Model 2 = Model 1 + adjusting laboratory parameters (serum white blood cells, hemoglobin, platelet, sodium and potassium)

Model 3 = Model 2 + mechanical ventilation, usage of vasoactive drugs, midazolam exposure, SOFA score and SAPSII score

CI confidence interval; OR: odds ratio; SOFA, Sequential Organ Failure Assessment, SAPSII, Simplified Acute Physiology Score-2; TyG, triglyceride-glucose index

### Subgroup analysis

**Fig. 3:**
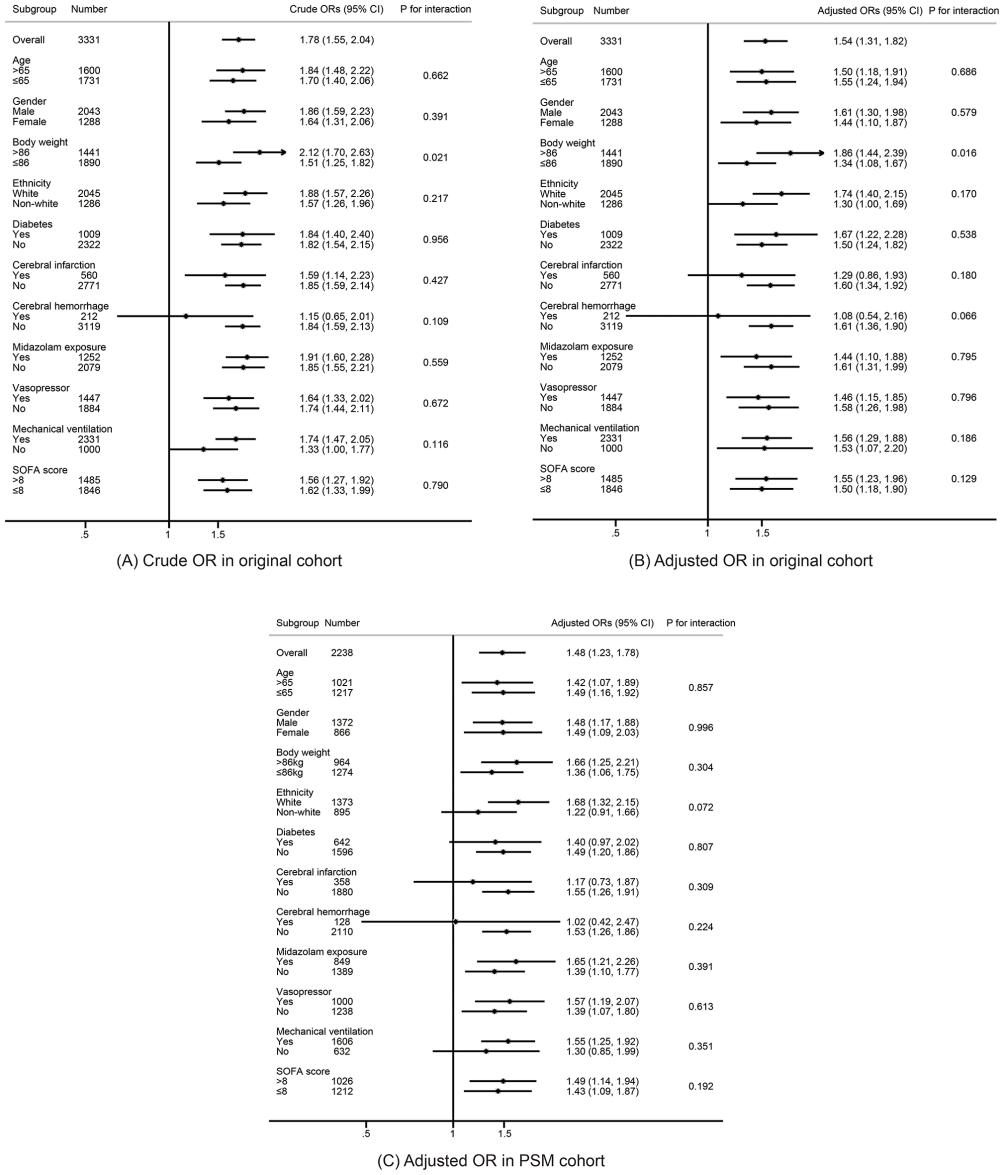
The forest plots of the subgroup analysis detecting the impact of TyG > 9.09 on developing delirium in different subgroups. (**A**-**B**) presents the crude and adjusted result in original cohort. (**C**) indicates the adjusted result in PSM matched cohort

Figure 3 presents the forest plot of the subgroup analysis used in the logistic regression analysis in both the original and PSM matched cohorts. The results of the subgroup analysis consistently supported that TyG > 9.09 was significantly associated with the increased risk of the development of delirium (all ORs > 1). Significant interaction effects were found for body weight × TyG (*P* for interaction < 0.05) in the original cohort, but none in the PSM matched cohort. We observed that the value of an elevated TyG level (> 9.09) for developing delirium was significantly lower in patients with a body weight ≤ 86 kg compared to those with a body weight > 86 kg, and the statistically significant associations were present in both subgroups in both the original cohort and the PSM cohort (all OR > 1, and *P* < 0.05). In septic patients with cerebral hemorrhage, there was still evidence of a positive association between an elevated TyG index and the risk of developing delirium, but no statistical significance was found in both the original and PSM cohorts (OR > 1, but *P* > 0.05).

## Discussion

Delirium is a common manifestation of neurological dysfunction in sepsis, leading to high mortality and poor prognosis. This retrospective study found that the increase in TyG index was associated with the risk of developing delirium. The RCS curves suggested a J-shaped relationship between TyG index and the risk of delirium in critical patients with sepsis, and the optimal cut-off value was recommended to be 9.09. TyG > 9.09 was significantly associated with the increased risk of developing delirium, even after baseline adjustment using the PSM method. Our research shows robust results in the subgroup analyses. In summary, our study shows the association between TyG index and the risk of developing delirium, indicating that increased TyG index may be expected to be a novel biomarker for delirium. To the best of our knowledge, this is the first study to describe the association between the TyG index and sepsis-associated delirium. Our research provides new insights into the early identification of sepsis-associated delirium, suggesting that the TyG index may be a potential biomarker for identifying delirium in patients with sepsis. Further research is needed to confirm our findings.

The biological mechanisms of delirium are complex, involving neuroinflammation, inadequate metabolism, brain barrier damage, neurotransmitter imbalance and impaired neuronal network connectivity [35]. It is known that systemic inflammation would cause neurological involvement. With an increasing in pro-inflammatory cytokines and oxidative, the systemic inflammation in sepsis may damage to the blood-brain barrier. And then microglia are activated as pro-inflammatory cytokines subsequently flow into the central nervous system [36, 37]. With the activation of microglia and astrocytes, it causes elevation of neuroinflammation and central nervous system (CNS) cytokines, further leading to delirium and pathological behavior, which is the main neuroinflammation mechanism of delirium [38, 39]. In addition, blood-brain barrier leakage triggers infiltration of peripheral monocytes in the brain, which is involved in the pathogenesis of CNS diseases [40, 41]. As a selectively permeable barrier, the intact blood-brain barrier function maintains homeostasis within the CNS and restricts the penetration of neurotoxic molecules, which is crucial for preventing CNS disorders such as delirium. Metabolic disorders represent another significant contributing factor to the occurrence of delirium, which can promote a CNS inflammatory response and damage to the blood-brain barrier. Excessive glucose metabolism can result in systemic inflammation and an increased generation of reactive oxygen species, while also mediating blood-brain barrier disruption [42]. Lipids represent the most abundant component of the CNS, including phospholipid bilayers and myelin sheaths, which collectively constitute over 50% of the brain’s dry weight [43]. In contrast to glucose, which exerts its effects during the initial stages of inflammation, lipids primarily regulate the inflammatory reaction during the later stages [44]. In the initial stages of disease, microglia respond to neural injury by engulfing a significant quantity of oxidized lipids and myelin phospholipid fragments. Nevertheless, as the engulfment of lipids increases, it gives a rise to secondary cytotoxic effects, and hyperlipidemia exasperates ischemic brain injury and mediates inflammation by promoting the upregulation of CD36 expression [45]. The impact of dysregulated glucose and lipid metabolism on delirium has attracted increasing attention, which can contribute to the occurrence of delirium through the interactions with other factors such as inflammatory responses, oxidative stress, and barrier damage.

Sepsis is caused by infection in the early stages and rapidly progress. It has been proved that infection is a crucial precipitating risk factor for delirium [46]. A recent meta-analysis study indicates an overall incidence rate of delirium at 23% [47]. while the incidence rate can reach up to 50-70% in patients with sepsis [10, 43]. Sepsis can lead to systemic multiple organ dysfunction and metabolic disorders, including a sharp increase of serum glucose and lipid levels. Various mechanisms participate in IR in sepsis. Specifically, pro-inflammatory cytokines can stimulate hepatic gluconeogenesis and alter insulin signaling through the production of toxins by Toll-like receptor 4 (TLR4) [48]. Additionally, it also plays an important role in IR by downregulating GLUT4 transcription and inhibiting the PI3K/Akt pathway [49]. Cytokine storms in sepsis can trigger immune mediated dyslipidemia. The high levels of endotoxins present in severe sepsis can inhibit the activity of lipoprotein lipase (LPL), causing an increased triglyceride in plasma [50]. Elevated plasma triglyceride level further amplifies inflammation and induces IR. In addition to the immune inflammatory response to infection, the neuroendocrine response of sepsis can also initiate excessive release of various hormones, leading to changes in metabolic status and participating in the occurrence of blood glucose and lipid metabolism disorders, even IR. Therapeutic and endogenous adrenaline and norepinephrine can trigger lipolysis, liver gluconeogenesis, glycogenolysis, and glycolysis, leading to elevated serum glucose and lipid levels [51]. Cortisol not only stimulates hepatic gluconeogenesis, but also induces IR, further exacerbating hyperglycemia [51, 52]. This induced IR is further amplified by inflammatory mediators, mainly including TNF- α, IL-1, IL-6, and C-reactive proteins [51].

The TyG index, as a clinical indicator of IR, has been shown certain value by multiple studies in predicting adverse prognosis of sepsis and related organ dysfunction [22, 53]. Rui Zheng et al. found that the TyG index is associated with an increased in-hospital mortality rate in critically ill patients of sepsis [22]. Yijiao Fang et al. reported that the level of TyG index is related to the occurrence and poor prognosis of sepsis associated acute kidney injury [53]. According to our findings, there are no relevant reports currently on the predictive value of TyG in delirium. As IR is closely associated with several cerebrovascular diseases, cognitive decline, sepsis, etc., the association between TyG index and delirium is worth exploring. This study found that elevated TyG is an independent risk factor of developing delirium, and further described a non-linear relationship between them, filling the gap in the use of TyG as an alternative IR indicator to predict delirium in sepsis. A recent study by Miao Sun et al., focusing on POD in elderly patients with type 2 diabetes, provided some support for the importance of our research [31]. They reported that patients with TyG index greater than the optimal cut-off value (8.678) have a higher risk of delirium by 59.0-66.8% compared to those without (OR 1.590–1.668 in different models) [31]. This may be due to the crucial role of insulin in neurosynaptic function, synaptic plasticity regulation, glucose uptake, and neuronal survival. Therefore, chronic IR can lead to dysfunction of the CNS [54]. Hong S et al. proposed an association between an increase in TyG index and a higher risk of cognitive decline in men [16]. In addition, the research by Xiaohuang Hua et al. has shown that a higher TyG index is associated with an increased risk of delirium and mortality in critically ill patients aged above 65 years [55]. However, in our study, no significant association between TyG index and mortality was found, which may be related to potential confounding factors, smaller sample sizes, and oversight of the impact of dynamic changes in TyG index on prognosis after delirium onset. As a retrospective observational study, our study lacked an exploration of the potential mechanisms by which IR affects delirium. We attempt to further explain the potential mechanism of IR as a predictive biomarker for SAE through literature review. Insulin receptors are widely expressed in various types of cells in the brain, which can be activated by insulin to promote neuronal survival and regulate various advanced brain functions. Under pathological conditions, IR is associated with cognitive impairment by affecting the CNS insulin signaling pathway. It is known that IR may inhibit the neuronal PI3-K/AKT pathway and enhance GSK3-β activation to increase tau protein hyperphosphorylation, a pathological indicator of cognitive decline, such as delirium [56]. Additionally, the abnormal deposition of Aβ protein has been a research hotspot in the pathogenesis of delirium for a long time. Insulin can inhibit formation of Aβ fibers and promote the internalization of Aβ oligomers, limiting neuronal binding and protecting synapses from the impact of Aβ oligomers [57]. As brain IR occurs, the degradation and clearance of Aβ protein are hindered, in the meanwhile, the abnormal sedimentation of Aβ protein stimulates neuroglial cells to release various inflammatory factors and produce oxygen free radical, inducing oxidative stress and activating cell apoptosis [58]. IR is mediated by tau protein hyperphosphorylation and the abnormal sedimentation of Aβ protein to affect the occurrence of delirium, which is also related to the occurrence of dementia. We already know that delirium is a risk factor for dementia. The research results of Jie Wang et al. suggested that preoperative IR affects the occurrence of postoperative delirium, which is associated with the impact of IR about the metabolism of AD biomarkers [17]. In addition, IR also damages to the endothelial function of macrophages, exacerbates atherosclerosis and dyslipidemia, which may be another factor leading to delirium.

Our study provides new evidence for the early identification of sepsis-associated delirium, suggesting that an elevated TyG has some value in predicting the development of delirium. However, as a retrospective study, the level of evidence is relatively low, and future high-quality prospective studies are needed to determine the clinical predictive value of the TyG index. It has been suggested that glucose dysregulation can lead to disruption of neuronal network in the CNS, which may result in the development of delirium [59, 60]. Changes in lipid metabolism patterns are also a key factor in the development of delirium [61]. Since TyG levels are influenced by glucose and triglycerides, it is worth exploring whether TyG-directed glucose and triglyceride regulation could be a preventive measure for delirium in septic patients. Furthermore, as TyG is a real-time parameter, and in particular is mainly influenced by glucose levels, the clinical value of a single TyG measurement may be limited. Dynamic monitoring of changes in TyG, such as using group-based trajectory models, will be a better approach.

Our findings need to be interpreted with caution, due to the following limitations. Firstly, as a retrospective study, the results are subject to the influence of confounding factors. Despite partial adjustment for confounders using logistic regression and PSM method, the presence of unknown confounders still affects the accuracy of our results. Secondly, although the MIMIC database provides a large sample size of critically ill patients, the findings are not necessarily generalizable due to the single-center nature of the study. Thirdly, our study only observed the association between TyG and delirium, and it remains unclear whether a causal relationship exists between them. Fourthly, as a clinical study, our investigation of the underlying mechanisms is limited. Further high-quality clinical and basic research is required to substantiate our findings.

## Conclusions

Our study indicates an association between the TyG index and the risk of developing delirium in patients with sepsis. The TyG index is a novel potential biomarker to predict the development of delirium in patients with sepsis and guide the early identification in clinical practice. Further high-quality clinical trials are needed to confirm our current findings.

### Electronic supplementary material

Below is the link to the electronic supplementary material.

Supplementary Material 1

## Data Availability

No datasets were generated or analysed during the current study.

## Abbreviations

CAM-ICU: the Confusion Assessment Method for the Intensive Care Unit
CI: Confident Interval
CNS: Central Nervous System
ICU: Intensive Care Unit
IQR: Interquartile Range
IR: Insulin Resistance
LOS: Length of Stays
MIMIC-IV: the Medical Information Mart for Intensive Care database
OR: Odds Ratio
POD: Postoperative Delirium
RCS: Restricted Cubic Spline
SAE: Sepsis associated encephalopathy
SOFA: Sequential Organ Failure Assessment
TyG: Triglyceride-glucose Index
VIF: Variance Inflation Factor
