# Supplementary material for: Association of triglyceride-glucose index and delirium in patients with sepsis: a retrospective study
Source: Lipids Health Dis. 2024 Jul 25;23:227. doi: 10.1186/s12944-024-02213-x (PMC11271053; doi:10.1186/s12944-024-02213-x)
Supplement: Supplementary file 1 — Supplementary Material 1 [file 12944_2024_2213_MOESM1_ESM.docx]

**Association of triglyceride-glucose index and delirium in patients with sepsis: a retrospective study**

Yipeng Fang^1^, Aizhen Dou^1^, Yuehao Shen^1^, Tianyu Li^1^, Haiying Liu^1^, Yan Cui^1,3,^*, Keliang Xie^1,2,^*

^1^Department of Critical Care Medicine, Tianjin Medical University General Hospital, Tianjin, 300052, China.

^2^Department of Anesthesiology, Tianjin Institute of Anesthesiology, Tianjin Medical University General Hospital, Tianjin, 300052, China.

^3^Department of Pathogen Biology, School of Basic Medical Science, Tianjin Medical University, Tianjin 300070, China.

**Corresponding Author:**

Prof. Keliang Xie, MD, PhD, Department of Critical Care Medicine, Tianjin Institute of Anesthesiology, Tianjin Medical University General Hospital, 154^th^ Anshan Road, Tianjin, 300052, China. Tel: +86–22–60814233, Fax: +86–22–27813550, Email: [xiekeliang2009@hotmail.com](mailto:xiekeliang2009@hotmail.com) or [mzk2011@126.com;](mailto:mzk2011@126.com;)

Prof. Yan Cui, MD, Department of Pathogen Biology, School of Basic Medical Sciences, Tianjin Medical University, No. 22, Qixiangtai Road, Heping District, Tianjin 300070, China. Tel: +86-22-83336816, E-mail: cuiyanbio45@163.com.

**Supplemental Material**

**Table S1** Variance inflation factor (VIF) before data conversion

**Table S2** Variance inflation factor (VIF) after data conversion

**Figure S1** Non-linear relationship between TyG index and the odds of developing delirium in PSM cohort.

Multicollinearity between variables was assessed by variance inflation factor (VIF). As shown in Table S1, sodium, TyG, potassium, age, hemoglobin, SAPSII, body weight and SOFA score had VIF > 10, indicating multicollinearity. By converting them to binary categorical variables based on median or mean, multicollinearity was eliminated (shown in Table S2).

**Table S1** Variance inflation factor (VIF) before data conversion

| Variable | VIF | 1/VIF |
| --- | --- | --- |
| Sodium | 196.77 | 0.005082 |
| TyG value | 179.09 | 0.005584 |
| Potassium | 35.91 | 0.027846 |
| Age | 28.64 | 0.034917 |
| Hemoglobin | 26.47 | 0.037782 |
| SAPSII | 18.99 | 0.052655 |
| Body weight | 14.52 | 0.068863 |
| SOFA | 13.78 | 0.072578 |
| Platelet | 5.88 | 0.170142 |
| White blood cell | 4.96 | 0.201582 |
| Mechanical ventilation | 4.59 | 0.217958 |
| Creatinine | 3.14 | 0.318491 |
| Male | 3.03 | 0.330232 |
| Vasoactive drugs | 2.93 | 0.341508 |
| Race white | 2.69 | 0.372211 |
| Hypertension | 2.46 | 0.406611 |
| Anemia | 2.30 | 0.436294 |
| Chronic kidney disease | 2.10 | 0.476121 |
| Midazolam | 1.91 | 0.524075 |
| Heart failure | 1.76 | 0.569131 |
| Diabetes | 1.74 | 0.573245 |
| Coronary heart disease | 1.72 | 0.580416 |
| Liver disease | 1.66 | 0.603788 |
| Pulmonary disease | 1.43 | 0.701303 |
| Cerebral infarction | 1.32 | 0.760256 |
| Malignant cancer | 1.31 | 0.765943 |
| Cerebral hemorrhage | 1.17 | 0.851903 |
| Mean VIF | 20.82 |  |

**Table S2** Variance inflation factor (VIF) after data conversion

| Variable | VIF | 1/VIF |
| --- | --- | --- |
| Platelet | 4.80 | 0.208269 |
| White blood cell | 4.73 | 0.211688 |
| Mechanical ventilation | 4.26 | 0.235245 |
| SOFA | 4.16 | 0.240594 |
| TyG value | 3.26 | 0.314069 |
| Creatinine | 2.88 | 0.347886 |
| Male | 2.84 | 0.365103 |
| Vasoactive drugs | 2.77 | 0.360871 |
| SAPSII | 2.74 | 0.365567 |
| Age | 2.63 | 0.386607 |
| Race white | 2.44 | 0.412335 |
| HT | 2.27 | 0.444264 |
| Potassium | 2.18 | 0.460048 |
| Hemoglobin | 2.18 | 0.46188 |
| Anemia | 2.13 | 0.470205 |
| Chronic kidney disease | 2.05 | 0.486928 |
| Midazolam | 1.89 | 0.530076 |
| Diabetes | 1.73 | 0.579508 |
| Heart failure | 1.72 | 0.582609 |
| Coronary heart disease | 1.71 | 0.583558 |
| Sodium | 1.68 | 0.597071 |
| Liver disease | 1.46 | 0.685658 |
| Pulmonary disease | 1.42 | 0.702141 |
| Cerebral infarction | 1.29 | 0.776754 |
| Malignant cancer | 1.25 | 0.801243 |
| Cerebral hemorrhage | 1.16 | 0.862151 |
| Mean VIF | 2.43 |  |

The RCS curve reveals that the J-shaped association between the TyG value and the odds of developing delirium in septic patients was still present in the PSM cohort (shown in Figure S1).


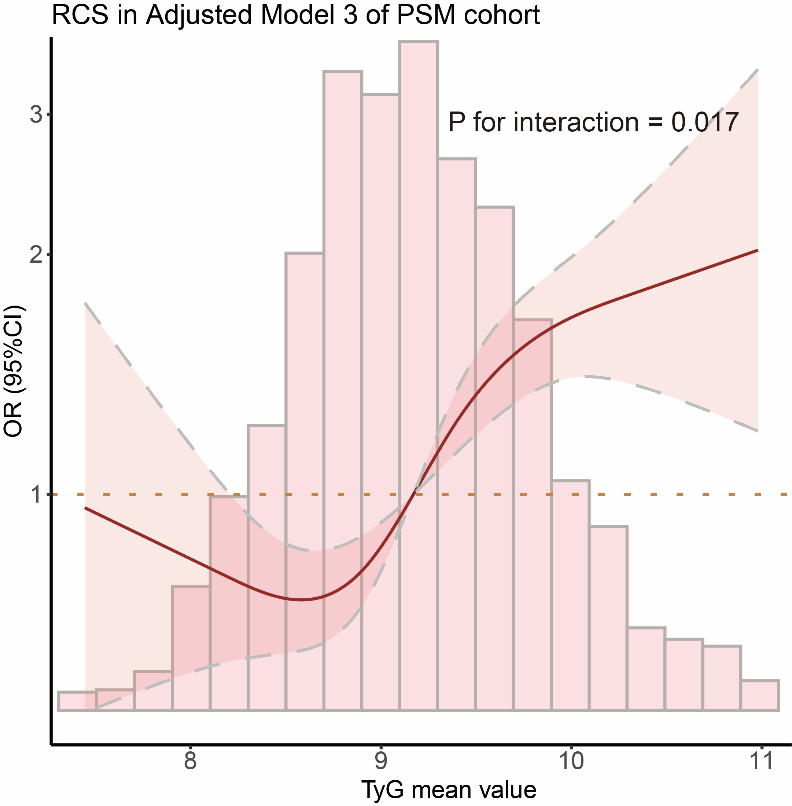


**Figure S1** Non-linear relationship between TyG index and the odds of developing delirium in PSM cohort.
